# Non-thermal plasma promotes hair growth by improving the inter-follicular macroenvironment

**DOI:** 10.1039/d1ra04625j

**Published:** 2021-08-17

**Authors:** Han-Jun Kim, Eun-Wook Choi, Eun-Ji Choi, Hyo-Sung Kim, Junggil Kim, Guangsup Cho, Heesu Kim, Seulgi Na, Jae Ho Shin, Sun Hee Do, Bong Joo Park

**Affiliations:** Department of Clinical Pathology, College of Veterinary Medicine, Konkuk University Seoul 05029 Republic of Korea shdo@konkuk.ac.kr +82 2 450 3706; Department of Bioengineering, Henry Samueli School of Engineering and Applied Sciences, University of California – Los Angeles Los Angeles CA 90095 USA; Terasaki Institute for Biomedical Innovation Los Angeles CA 90024 USA; R&D Center, Prostemics Co., Ltd Seoul 04778 Republic of Korea; Department of Electrical Biological Physics, Kwangwoon University Seoul 01897 Republic of Korea parkbj@kw.ac.kr +82 2 940 8629; Department of Chemistry, Kwangwoon University Seoul 01897 Republic of Korea; Institute of Biomaterials, Kwangwoon University Seoul 01897 Republic of Korea

## Abstract

Non-thermal plasma (NTP) is widely used in the disinfection and surface modification of biomaterials. NTP treatment can regenerate and improve skin function; however, its effectiveness on hair follicle (HF) growth and its underlying mechanisms need to be elucidated. Herein, we propose an air-based NTP treatment, which generates exogenous nitric oxide (eNO), as a therapeutic strategy for hair growth. The topical application of air-based NTP generates large amounts of eNO, which can be directly detected using a microelectrode NO sensor, in the dermis of mouse dorsal skin. Additionally, NTP-induced eNO has no cytotoxicity in normal human skin cells and promotes hair growth by increasing capillary tube formation, cellular proliferation, and hair/angiogenesis-related protein expression. Furthermore, NTP treatment promotes hair growth with adipogenesis and activation of CD34^+^CD44^+^ stem cells and improves the inter-follicular macroenvironment *via* increased perifollicular vascularity in the mouse hair regrowth model. Given the importance of the hair follicle (HF) cycle ratio (growth *vs.* regression *vs.* resting) in diagnosing alopecia, NTP treatment upregulates the stem cell activity of the HF to promote the anagen : catagen : telogen ratio, leading to improved hair growth. We confirmed the upregulation of increasing Wnt/β-catenin signaling and activation of perifollicular adipose tissue and angiogenesis in HF regeneration. In conclusion, these results show that the eNO from NTP enhances the cellular activities of human skin cells and endothelial cells *in vitro* and stem cells *in vivo*, thereby increasing angiogenesis, adipogenesis, and hair growth in the skin dermis. Furthermore, the results of this study suggest that NTP treatment may be a highly efficient alternative in regenerative medicine for achieving enhanced hair growth.

## Abstract 2

Non-thermal plasma (NTP) is widely used in the disinfection and surface modification of biomaterials.

## Graphical abstract

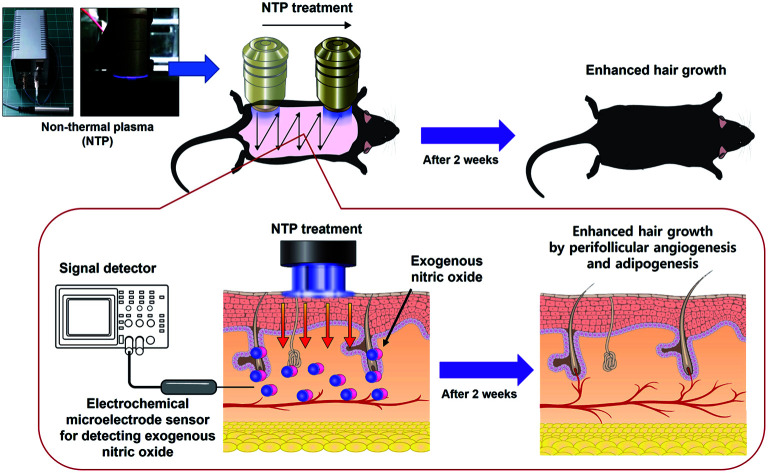

## Introduction

Hair loss can have a negative impact on an individual's psychological well-being and can be a socio-economic burden. Studies on the mechanisms of hair follicle (HF) regeneration and growth regulation provide opportunities for a variety of clinical drugs and non-medical and cosmetic interventions; however, to date, no approach has provided a complete solution. There exist a limited number of FDA-approved hair loss treatments. Minoxidil is one of the few hair loss drugs approved by the FDA and was initially developed as a treatment for high blood pressure.^1–4^ Minoxidil induces peripheral vasodilation and increases blood flow by stimulating vascular endothelial growth factor (VEGF) expression.^5–8^ The efficacy of minoxidil varies depending on the individual, and when its use is discontinued, its hair-growth effect does not last, and eventually, hair loss recurs. Additionally, minoxidil may cause side effects such as itching, dandruff, dryness, and allergic contact dermatitis. Accordingly, a novel treatment strategy for alopecia is needed to overcome these problems.^4,9–11^

Recently, various laser technologies have emerged as an alternative treatment for hair loss patients. Certain laser therapies are widely used in the treatment of alopecia, and such laser devices have become very popular owing to their commercial availability at clinics. However, some laser treatments generally require a considerable amount of time for wound healing and hair growth because the lasers induce microtrauma/microscopic injuries or destroy the skin barriers, although the damages trigger a favorable wound healing environment that induces hair growth. Moreover, there are still limitations in understanding the mechanisms of hair regrowth with laser technologies, and it is difficult to select the treatment parameters compared to those in the case of other technologies for hair loss treatment. Furthermore, some lasers, such as fractional lasers, are relatively more expensive, and some treatments have been shown to induce side effects along with burns.^12–16^

Non-thermal plasma (NTP) is also an alternative technology and a promising therapeutic approach for hair regrowth. Plasma is one of the four fundamental states of matter and has been widely used in the disinfection and surface modification of biomaterials. The potential for the clinical use of NTP in the treatment of skin diseases such as chronic wounds, acne, and pressure ulcers in the dermatological field has been reported.^17–19^ Recent studies have provided evidence of the anti-aging effect and safety of NTP. NTP has also been reported to significantly increase the level of bioactive factors in mouse skin, including TGF-α, TGF-β, VEGF, GM-CSF, and EGF.^20^ Choi *et al.* also demonstrated that repeated NTP treatment stimulates epidermal cell growth and accelerates wound healing by downregulating E-cadherin and stimulating β-catenin.^20^ These studies clearly indicate that NTP can be an effective tool for skin dermal tissue remodeling and the healing of skin wounds.

In addition, NTP produces reactive oxygen species (ROS) and nitrogen species (RNS), including hydrogen peroxide, superoxide, hydroxyl radicals, and nitrogen oxide (NO).^21–26^ These ROS generally exhibit strong oxidative properties, which can induce modifications of biological molecules such as lipids, proteins, and DNA/RNA.^21–26^ However, NO can act as a signal molecule for cellular functions and as a regulator of cellular activity triggering immune-deficiencies, cell proliferation, and cellular activation inducing angiogenesis, phagocytosis, and collagen synthesis.^22^

Therefore, we focused on the exogenous nitric oxide (eNO) generated by NTP treatment in this study, because NO exhibits various positive functions as described above and plays an essential role in the physiology of the skin.^23,25^ Accordingly, we directly measured the amount of eNO in media *in vitro* and in mouse skin *in vivo* using an electrochemically fabricated microelectrode NO sensor, and also investigated the effects of eNO on *in vitro* cell functions such as cytotoxicity, proliferation, and differentiation using four types of normal human skin cells: keratinocytes, dermal fibroblasts, dermal papilla cells, and endothelial cells. We further verified the hair-growth-promoting effects *in vivo* along with the enhancement of perifollicular angiogenesis and adipogenesis using hair-cycle-synchronized C57BL/6 mice.

## Materials and methods

### Fabrication and characterization of NTP

**Fig. 1:**
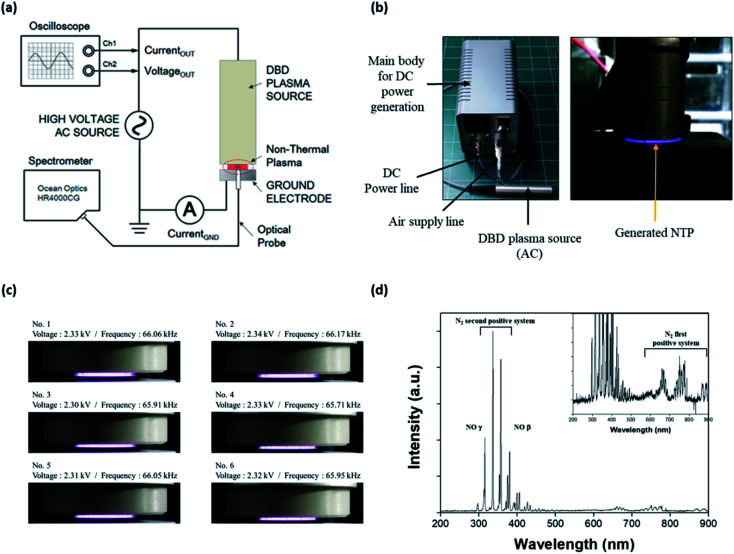
NTP and optical emission spectroscopy analysis. (a) Schematic illustration of the experimental configuration for NTP. (b) The NTP device and generated NTP. (c) Stability of the generated plasma at 2.30–2.34 kV. (d) Optical emission spectroscopy (OES) of NTP. The OES of NTP was conducted using a spectrometer (HR4000CG-UV-NIR). NTP: non-thermal plasma.

An NTP device based on a dielectric barrier discharge (DBD) was fabricated, as shown in Fig. 1a; it consisted of the main body for supplying DC power to the NTP source, a DC power line, an air supply line, and a DBD plasma source comprising three parts: a dielectric layer (a disk of diameter 10 mm and thickness 0.5 mm), a voltage-applied copper electrode (of diameter 8 mm), and a gas nozzle. The working gas was air delivered using a rotary diaphragm pump (2 L min^−1^ free flow rate) and injected from the side to the center of a disk on the device. The AC power supply in the DBD plasma source was a commercially available LCD backlight inverter operated at 60 kHz. The applied AC output voltage was 1.0–3.7 kV_RMS_, and the power consumption was less than 10 W. The temperature of the plasma device contact area at a maximum treatment time of 5 min was below 40 °C.

The optical emission spectrum (OES) of the NTP was measured as a function of time using a portable Near-InfraRed (NIR) spectrometer (HR4000CG-UV-NIR, Ocean Insight, Orlando, FL, USA) (Fig. 1b). The OES collected from the surface light of the plasma panel was measured to analyze the ROS distribution of the plasma. The spectroscopic analyzer system consisted of an optical fiber (QP400-2-SR) using a biconvex lens with a focal length of 101 mm and a spectrophotometer with a linear CCD detector array. Instrumental line broadening was measured at approximately 0.75 nm using a cold argon–mercury lamp (CAL-2000, Avantes, Apeldoorn, Netherlands). The optical fiber was scanned in the horizontal and vertical directions along with the plasma panel by using a 2-axis optical micrometer to identify the strong emission signal.

### Quantification of eNO generated by NTP treatment in media

To detect eNO in distilled water (DW) and the cell culture media, a Griess reagent chemical method was used to determine the level of nitrites and nitrates (NO_*x*_^−^) generated by the NTP. Briefly, 1 mL each of DW and Dulbecco's modified eagle medium (DMEM, Welgene, Inc. Gyeongsan-si, Korea) with 10% fetal bovine serum (FBS, Welgene, Inc.) (DMEM 10% FBS) was exposed to NTP for different treatment periods (0, 10, 20, and 30 s). Next, 100 μL of NTP-treated DW and DMEM 10% FBS or standard sodium nitrite (NaNO_2_), as well as 50 μL of sulfanilamide solution, was added to a 96-well plate; 50 μL of *N*-1-napthylethylenediamine dihydrochloride solution was added to each of the wells. After the reaction had continued for 10 min at room temperature, optical absorbance was measured using a multimodal microplate reader (Cytation 3, BioTek Instruments, Inc. Winooski, VT, USA). NO concentrations were calculated with reference to the standard curve of NaNO_2_ generated from known concentrations.

### Fabrication and characterization of xerogel-derived microelectrode to detect eNO generated by NTP treatment *in vitro* and *in vivo*

To detect eNO in media and the dermis of mouse skin using an electrochemical method, a xerogel-derived microelectrode was fabricated and used, as previously described.^28–31^ Briefly, to fabricate a Pt microelectrode with a cone-shape,^28,30,31^ Pt wire (127 μm in diameter) was electrochemically etched for 1 min with a 5 V alternating current (AC) (60 Hz) by using a bipotentiostat (CH Instruments Model 760D, Austin, TX, USA) after immersing in an etching solution (1.2 M calcium chloride in water/acetone, 2 : 1, v/v). The etched Pt wire was inserted in a borosilicate glass capillary (outer and inner diameters of 600 μm and 550 μm, respectively) (A-M Systems, Carlsborg, WA, USA), shielded around the Pt wire with a gas torch, and rinsed with DW. The Pt electrode was platinized to achieve mesoporous platinum black (Pt-B) on the surface of the Pt electrode in chloroplatinic acid hexahydrate (3%) and lead(ii) acetate trihydrate (0.03%) solution (wt/wt in water).

As a reference electrode, an Ag/AgCl wire was fabricated by immersing a silver (Ag) wire (of diameter 250 μm) in 0.3 M FeCl_3_ solution for 15 min. Then, the Ag/AgCl wire was coiled around the borosilicate glass capillary.

To eliminate interference from common biological species (*i.e.*, nitrite, uric acid, ascorbic acid, acetaminophen, and carbon monoxide), the fabricated Pt–B/Pt electrode was modified with a perfluorinated xerogel-derived gas-permeable membrane in a silane sol solution to measure the NO *in vitro* and *in vivo* as previously described.^28,31^ The performance of the fabricated NO microsensor was evaluated by amperometric measurement using a Compactstat (Ivium Technology, Netherlands).

### Amperometric measurement of eNO generated by NTP treatment in media and mouse skin

To detect eNO in DW and the cell culture media using an electrochemical method, agarose (1.25%, Sigma-Aldrich, St. Louis, MO, USA) with a low gelling temperature was used to mimic skin *in vitro*. The concentrations of eNO generated by the NTP were quantified in the agarose containing DW and DMEM 10% FBS by using the fabricated electrochemical NO sensor, which was inserted into the agarose before it hardened. NTP treatments with different treatment periods (0, 10, 20, and 30 s) were conducted on the agarose, and the eNO measurements were performed.

To measure the eNO generated by NTP in the dorsal skin of the mouse quantitatively, male C57BL/6 mice (8 week-old) were used. The hair on the dorsal skin of the mice was shaved repeatedly using a clipper and completely removed using the commercial hair removal cream Veet (Reckitt Benckiser, Berkshire, UK). After anesthetizing the mice (*n* = 3), the fabricated electrochemical NO sensor was inserted into the dorsal skin of each mouse and stabilized. Next, NTP treatment (2.3 kV for 30 s) was performed on the dorsal skin of the mouse, and the generated eNO was measured.

### Cytotoxicity and proliferation assays in normal human skin cells

Human keratinocyte cells (HaCaTs) and normal human dermal fibroblasts (nHDFs) were maintained in DMEM with 10% FBS and 1% antibiotics (penicillin–streptomycin). Human dermal papilla cells (HDPCs) were cultured in an HDPC basal medium (CB-HDP-BM CEFO Co.) containing a 2% growth medium (CB-HDP-GM, CEFO Co.) and 0.05% antibiotic solution. For the cytotoxicity assay, all the pre-cultured cells were seeded (1.5 × 10^5^ cells per mL) into 35 mm cell culture dishes. The cells were further cultured at 37 °C and 5% CO_2_ in an incubator to allow for full cell attachment and spreading. After 24 h, the cells were exposed to NTP for 30 s after exchanging the media for new media and allowing further incubation for 24 h to confirm the cytotoxicity of the NTP treatment. The viability of each cell line was detected using a cell viability detection kit (Cell Counting Kit-8 (CCK-8); Dojindo Laboratories, Kumamoto, Japan). Each cell line was incubated with a CCK-8 reagent for 15 min at 37 °C in the dark after exchanging the media. Next, each solution in the 6-well plates was transferred into a 96-well plate to detect the optical absorbance at 450 nm using a multimodal microplate reader (Cytation 3). The cell viability was calculated as the percentage of viable cells in the treated group divided by the percentage of viable cells in the negative control group.

For the cell proliferation assay after the NTP treatment, pre-cultured HaCaTs, nHDFs, and HDPCs were plated (0.5 × 10^4^ cells per mL) in 35 mm cell culture dishes and incubated for 24 h. Next, all cells were further incubated for 5 days after exposure to minoxidil (1 μM) as a positive control, and NTP (2.3 kV for 30 s) was applied. On day 5, cell viability was evaluated using a CCK-8 assay reagent as described above.

### *In vitro* capillary tube formation by NTP treatment

For capillary tube formation *via* NTP treatment, human umbilical vein endothelial cells (HUVECs) were supplied by Cell Engineering For Origin (CEFO Co. Seoul, Korea) and cultured in a HUVEC basal medium (CB-HUVEC-BM, CEFO Co.) containing 7% HUVEC growth medium (CB-HUVEC-GM, CEFO Co.) and 0.05% antibiotic solution at 37 °C and 5% CO_2_. HUVECs between passages 5 and 6 were used for the capillary tube formation assay with NTP treatment, as previously described.^32^ Briefly, 180 μL of Matrigel (growth factor reduced; BD Biosciences, Franklin Lakes, NJ, USA) was inserted into each well of a 24-well μ-plate (ibidi GmbH, Germany) and the 24-well plate was incubated at 37 °C for 30 min to allow for hardening of the Matrigel. To better visualize the HUVECs and capillary tube formation through fluorescence imaging, the pre-cultured HUVECs-stained with calcein AM (2 μg mL^−1^) for 30 min—were seeded at 0.4 × 10^5^ cells per well into the Matrigel treated 24-well plate with 1 mL of CB-HUVEC-GM, and NTP (2.3 kV for 30 s) was applied to the plates. Minoxidil (1 μM) was used as a positive control, and NO–PBS solution (190 nM) was also used to compare the effects of NO and NTP treatment. The NO–PBS solution was diluted from a NO solution of 1.9 mM prepared by purging with NO (99.99%) gas for 30 min after removing any oxygen in the DPBS by purging with nitrogen gas for 30 min. Next, the HUVECs were incubated in a live cell imaging incubator (Lionheart FX; BioTek Instruments, USA) to analyze the new capillary tubes after transfer into the Matrigel-coated 24-well plate. After 8 h, fluorescence images of HUVECs were collected using a 4× objective lens and fluorescence optics (excitation/emission at 469/525 nm for GFP), and a 5 × 5 array (25 images) of each well was taken. The images were analyzed using Gen5 software (BioTek Instruments) after stitching all the images into one image. The number of capillary tubes, branch points, and the total length of the capillary tubes was analyzed using ImageJ software (NIH, Bethesda, MD, USA).

### Antibody microarray analysis of protein expression related to hair growth and angiogenesis in normal human skin cells

To investigate the differential protein expression following NTP treatment, a Signaling Explorer Antibody Array kit (SET100) was purchased from Fullmoon Biosystems, Inc. (Sunnyvale, CA, USA) and antibody microarray assays were carried out using HaCaTs, nHDFs, and HDPCs according to the manufacturer's protocol. Briefly, total proteins were extracted from the three cells incubated for 24 h after NTP treatment by using a protein extraction buffer (Fullmoon Biosystems) containing 1% protease inhibitor cocktail (Sigma-Aldrich, St. Louis, MO, USA), 1% phosphatase inhibitor cocktail (Sigma-Aldrich), and lysis beads (Fullmoon Biosystems). After protein extraction, glass slides for antibody microarrays were incubated on a shaking incubator for 45 min after coating with 1358 antibodies. After treatment with a blocking solution, the slides were thoroughly washed with Milli-Q grade DW, after which bound biotinylated proteins were detected using Cy3-labeled streptavidin (1 : 1000 dilution, GE Healthcare, Little Chalfont, UK). After 20 min of incubation, the slides were washed and completely dried, and then scanned with a GenePix 4100A scanner (Molecular Devices, Sunnyvale, CA, USA). The scanned images were quantified using GenePix 7.0 software (Molecular Devices). Numeric data were analyzed using Genowiz 4.0 (Ocimum Biosolutions, Hyderabad, India), and protein data information was annotated using the UniProt database (available at www.uniprot.org).

### Enzyme-linked immunosorbent assay (ELISA) for β-catenin and IGF-1 in HDPCs

To confirm the protein expression in HDPCs following the NTP treatment, the protein expression levels of β-catenin and IGF-1 were measured in the HDPCs cultured medium after NTP treatment at 2.3 kV for 30 s using the enzyme-linked immunosorbent assay (ELISA) kit (R&D System, USA). Samples, including positive control and NO-PBS groups, were loaded on a 96-well plate containing a carbonate coating buffer for 24 h at 4 °C. Next, the carbonate coating buffer in the 96-well plate was removed and further incubated for 2 h at 37 °C after adding a blocking buffer (5% skim milk in PBST). After blocking, primary antibodies against β-catenin and IGF-1 and secondary antibodies were added and incubated for another 2 h at 37 °C. All antibodies were washed out, and a substrate (*o*-phenylenediamine dihydrochloride/hydrogen peroxide) was added and incubated for 30 min at 37 °C in dark conditions. The reaction was stopped by adding sulfuric acid for 10 min at 37 °C in dark conditions. The optical density of each concentration was examined by measuring its absorbance at 490 nm using a multimodal microplate reader (Cytation 3). All samples were assayed in quadruplicate experiments, and the relative protein expression level was calculated as the percentage of protein expression in the NTP treated group divided by the percentage of the negative control (NC) group.

### *In vitro* hair-growth promotion study

HDPCs (6 × 10^5^ cells per well) were seeded on Aggrewell 800 plates (Stemcell Technologies Inc. Seattle, WA, USA) and incubated for 24 h. Next, the HaCaTs (1.2 × 10^6^ cells per well) were seeded in an HDPC spheroid in the upper compartment and incubated overnight. Five μM NO–PBS, which was diluted from a NO solution (1.9 mM), or 1 μM minoxidil (Sigma-Aldrich) was added to the medium of co-cultured HaCaT + HDPC (Co-HaCAt + HDPC) and incubated for 3 days. Each experiment was conducted in triplicate.

After harvesting the cells, the total RNA was isolated using a Qiazol lysis reagent (Qiagen, Valencia, CA, USA) and 1 μg of the isolated RNA was reverse transcribed into complementary DNA (cDNA) and used in quantitative (qPCR) assays using a SYBR Green PCR Master Mix (Thermo Fisher Scientific, Seoul, Korea) in a QuantStudio 3 Real-Time PCR System (Thermo Fisher Scientific, Seoul, Korea). The reactions (20 μL) comprised 2 μL of diluted cDNA, 2 μL of each primer, 10 μL of 2× SYBR Green PCR Master Mix, and 6 μL of RNase-free water. The sequences of the oligonucleotide primers used in the qPCR assays are shown in Table 1. Thermocycling conditions were 50 °C for 2 min, 95 °C for 10 min, 40 cycles of 95 °C for 15 s, and 60 °C for 1 min. All samples were assayed in triplicate, and mRNA levels were calculated using the 2^−ΔΔCt^ method.

**Table 1:** Sequences of human primers used for real-time PCR

Target gene	Source	Sequence	Predicted length (bp)
GAPDH	NM_002046	F: 5′-GTCTCCTCTGACTTCAACAGCG-3′	131
		R: 5′-ACCACCCTGTTGCTGTAGCCAA-3′	
β-Catenin	NM_001904	F: 5′-CACAAGCAGAGTGCTGAAGGTG-3′	146
		R: 5′-GATTCCTGAGAGTCCAAAGACAG-3′	
ALP	NM_000478	F: 5′-TCTCTTCGAGCCAGGGGACA-3′	82
		R: 5′-CCACCACCATCTCGGAGAGT-3′	
Wnt5a	NM_003392	F: 5′-TACGAGAGTGCTCGCATCCTCA-3′	123
		R: 5′-TGTCTTCAGGCTACATGAGCCG-3′	
Wnt10b	NM_003394	F: 5′-AATTCTGGAGCCTTCCAGCC-3′	260
		R: 5′-TCACACAGCACATAGCAGCA-3′	
BMP2	NM_001200	F: 5′-ACTCGAAATTCCCCGTGACC-3′	146
		R: 5′-CCACTTCCACCACGAATCCA-3′	
BMP4	NM_001202	F: 5′-ATGTGGGCTGGAATGACTGG-3′	197
		R: 5′-ATGGCACTCAGTTCAGTGGG-3′	
TGF-β1	NM_000660	F: 5′-TACCTGAACCCGTGTTGCTCTC-3′	122
		R: 5′-GTTGCTGAGGTATCGCCAGGAA-3′	
TGF-β2	NM_001135599	F: 5′-AAGAAGCGTGCTTTGGATGCGG-3′	151
		R: 5′-ATGCTCCAGCACAGAAGTTGGC-3′	

### *In vivo* hair-growth promotion study

Male C57BL/6 mice (eight-week-old) were obtained from YoungBio (Seongnam, Korea). The mice were housed under controlled conditions at a temperature of 23 ± 2 °C, humidity of 50% ± 5%, and light-dark cycle of 12 h. All experimental procedures were performed in compliance with protocols of all appropriate regulatory standards and were approved by the Institutional Animal Care and Use Committee of Konkuk University (KU16179; date of approval, 31 October 2016).

**Fig. 2:**
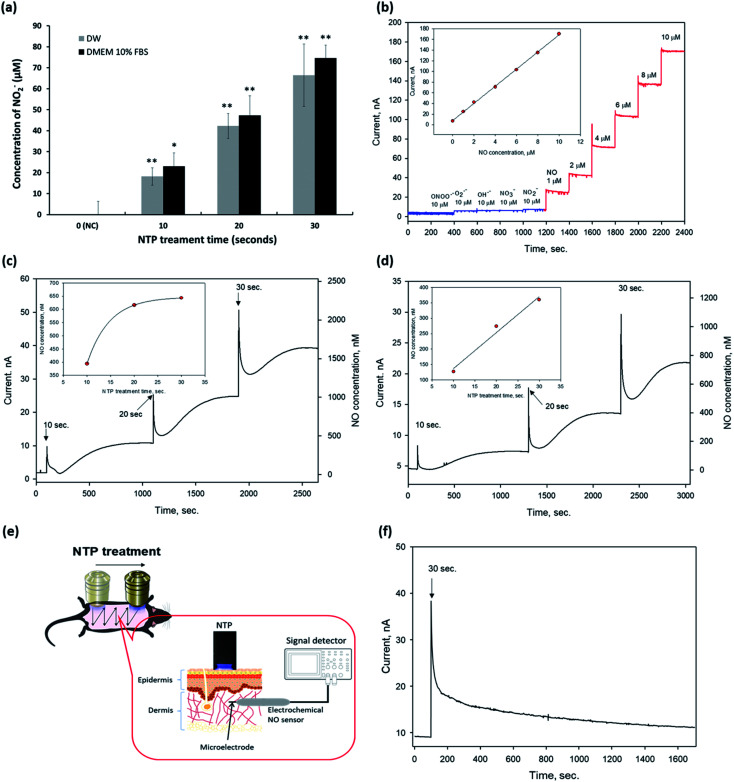
Detection of exogenous nitric oxide (eNO) generated by NTP treatment. (a) Quantitative analysis of NO_2_^−^ in DW and DMEM 10% FBS using the Griess reagent kit. Data are represented as the mean ± SD (*n* = 4, *p < 0.005, **p < 0.001 *vs.* NC). (b) Characterization of the NO microsensor modified with a perfluorinated xerogel-derived gas-permeable membrane. Dynamic response of a nitric oxide sensor using a Pt–B/Pt microelectrode modified with a perfluorinated xerogel membrane toward a series of interfering species (blue) and nitric oxide (red). Currents were detected at an applied potential of +0.8 *vs.* Ag/AgCl in PBS (0.01 M, pH 7.4). The inset represents the calibration curve. Quantitative analysis of eNO in (c) DW and (d) DMEM 10% FBS after plasma treatment (2.3 kV) for 10, 20, and 30 s, respectively. (e) Schematic of generated exogenous NO (eNO) detection using an electrochemical microelectrode NO sensor in the dermis of the mouse dorsal skin (*in vivo*). (f) Quantitative analysis of eNO in the dermis of the mouse skin (*in vivo*) after plasma treatment (2.3 kV) for 30 s. DW: distilled water, DMEM: Dulbecco's modified eagle's medium, FBS: fetal bovine serum, NC: negative control, and NTP: non-thermal plasma.

The hair on the dorsal skin of the mice was shaved repeatedly using an electric clipper and completely removed using the commercial hair removal cream Veet (Reckitt Benckiser, Berkshire, UK) to synchronize the HF cycle.^33–35^ The experimental group was randomly divided into three groups (*n* = 5/group): the negative control (NC); positive control (PC) with 200 μL of 3 wt% minoxidil topical application; and non-thermal plasma (NTP) treatment (2.3 kV for 30 s). After removing the hair, the entire dorsal skin was treated in a zigzag manner using the NTP as shown in Fig. 2(e) and 6(a), and the treatment was performed three times per week for four weeks, and minoxidil was applied five times per week.

### Histological analysis

The skin tissues were fixed with 10% neutral-buffered formalin (BBC Biochemical, Mount Vernon, WA, USA). The tissues embedded in paraffin were sectioned at 4 μm thickness, followed by staining with hematoxylin and eosin for histological analysis. The number and diameter of HFs were quantified in at least three fields per mouse at 100× magnification. The thickness of the adipocyte layer was measured in at least three fields per mouse at 40× magnification. The number and diameter of vessels were quantified in multiple fields at 100× magnification. The vessel diameter was measured in transversely cut vessels, and 30 vessels were measured for each group. The vessel density was determined by the area covered with vessels at 100× magnification.

### Immunohistochemistry and immunofluorescence

For immunohistochemistry analysis, the sections were deparaffinized and boiled in a citrate buffer solution. The sections were stained using standard methods with rabbit anti-CD31 (PECAM-1, Santa Cruz Biotechnology, Inc. 1 : 100) as the primary antibody. The sections were incubated using a Vectastain Elite ABC-Peroxidase kit (Vector Laboratories, Burlingame, CA, USA), visualized using a Vector SG (Vector Laboratories), and counterstained with nuclear fast solutions (Vector Laboratories). For immunofluorescence, the sections were deparaffinized and boiled in a citrate buffer solution. They were stained using standard methods with rabbit anti-IGF-1 (dilution 1 : 100, Abcam, Cambridge, UK) and mouse anti-β-catenin (dilution 1 : 100, Abcam) as the primary antibodies. The sections were incubated with fluorescein isothiocyanate-conjugated goat anti-mouse and Texas red-conjugated goat anti-rabbit secondary antibodies (dilution 1 : 100, Santa Cruz Biotechnology, Inc.). The sections were fixed with fluoromount (Vector Laboratories) and viewed under a fluorescence microscope (Leica Microsystems, Wetzlar, Germany).

### RNA *in situ* hybridization

RNA *in situ* hybridization was performed using an RNAscope 2-plex Detection Kit (Chromogenic, Advanced Cell Diagnostics, Hayward, CA, USA). CD34, CD44, and LGR5 were procured from Advanced Cell Diagnostics.

### Statistical analysis

All values obtained in the analysis are represented as the mean ± standard deviation (SD) of quadruplicate experiments. Statistical analysis of *in vitro* experiments was performed using SPSS Statistics (IBM SPSS Statistics, NY, USA). Statistical analysis of *in vivo* experiments was performed using Instat 3 (GraphPad Software, La Jolla, CA, USA). Multiple comparisons for all data were analyzed using the one-way analysis of variance (ANOVA) method and a Bonferroni *post hoc* paired comparison test. Statistical differences were considered as significant at *p* < 0.05.

## Results and discussion

### Characterization of non-thermal plasma

NTP is an easy-to-use, non-invasive method that can also be developed as a wearable device, for example, as an attachable pad.^32^ Advances in NTP devices and their application could provide significant benefits in terms of the control of various skin conditions, including hair loss. To apply NTP to the *in vitro* and *in vivo* hair loss model, we first fabricated a DBD NTP device and evaluated its stability and OES. As shown in Fig. 1c, the generated plasma was very stable over the 2.30–2.34 kV range. The electrical and electromagnetic safety of the NTP device was further tested by a specialized agency (Korea Testing Certification: KTC), and it was confirmed that the NTP device was safe for dermatological applications (data not shown).

NTP has been reported to produce ROS and RNS, including hydrogen peroxide, superoxide, hydroxyl radicals, and NO.^21–26^ These NTP-driven ROS and RNS exhibit antimicrobial activity and can regulate tissue repair and angiogenesis.^36,37^ The surface light of the DBD NTP panel was investigated using OES analysis to identify atomic and molecular nitrogen as well as other constituent species produced by the NTP. As shown in Fig. 1d, the emission spectrum observed on the plasma panel was collected over a range of 200–900 nm. The emission lines and bands were identified as described previously.^38^ Most emission lines showed transitions of molecular nitrogen. The first positive system (N_2_ (B^3^Π_g_–A^3^Σ_u_^+^) transitions) was in the 500–900 nm range (*e.g.*, 680 and 750 nm). High-intensity N_2_ second-positive atomic emission lines (C^3^Π_u′_–B^3^Π_g_) at 315.3, 336.7, 357.4, and 379.8 nm were identified. Additionally, this spectrum showed the NO β system (NO (B^2^Π–X^2^Π) transitions) in the 300–400 nm range, and the γ system (NO (A^2^Σ^+^–X^2^Π) transitions) in the 200–300 nm range.

This result shows that the formation of N_2_ long-lived metastable states as reservoirs of energy promote plasma chemical reactions. By contrast, low-intensity NO and OH emission lines were in the 250–450 nm range. These results show that the chemical and physical reactions produced various ROS such as NO, OH, and O *via* the plasma panel. Among them, NO was the most highly secreted ROS in response to our DBD-based NTP. Therefore, we focused on NO, because it is a well-known signaling molecule related to many physiological and pathological processes in the body, and it can play a key role in skin physiology.^18^

### *In vitro* and *in vivo* eNO detection

Next, we detected eNO concentrations in the NTP-applied media *in vitro* and the dermis of the mouse skin *in vivo* to verify the amount of eNO produced by NTP using two methods: a chemical and an electrochemical method.

First, we investigated the exogenous NO_2_^−^ generated by NTP in DW and the cell culture medium by using Griess reagents (a chemical method), as shown in Fig. 2a. NTP treatment significantly increased the exogenous NO_2_^−^ depending on the NTP treatment time in both, and the amount of generated NO_2_^−^ was approximately 18 ± 4.1, 42 ± 5.9, and 66 ± 14.8 μM in DW and 23 ± 6.3, 47 ± 6.4, 75 ± 9.3 μM in the cell culture medium (DMEM 10% FBS) after 10, 20, and 30 s of NTP treatment, respectively. From this result, we confirmed that the NTP treatment in DMEM 10% FBS tended to slightly increase the amount of exogenous NO_2_^−^, as compared to the group treated in DW. However, as the Griess reagent method comprehensively measures the levels of nitrite and nitrate or total NO, there is a limit to its ability to accurately determine the amount of NO generated by NTP treatment. Therefore, an electrochemical method using an electrochemical NO microsensor was used to accurately measure the amount of eNO generated by NTP treatment *in vitro* and *in vivo*.

For the electrochemical method, the fabricated NO microsensor, which has a membrane selectively permeable toward NO, was characterized by the sensitivity of 16.6 nA μM^−1^, a detection limit of 5 nM (S/N = 3), and response time of <3 s (*t*_95%_), along with a negligible response toward interfering species (at 10 μM for each species) such as peroxynitrite (ONOO^−^), superoxide (O_2_˙^−^), hydroxyl radical (OH˙), nitrate (NO_3_^−^), and nitrite (NO_2_^−^) (Fig. 2b).

To measure eNO, the NO microsensor was inserted into the 1.25% agarose for *in vitro* and the dermis of mouse skin (∼100–300 μm in depth) for *in vivo*. Next, NTP treatment was applied to the agarose and the mouse dorsal skin to measure the eNO generated by the NTP treatment. The Compactstat analysis (Ivium Technology, Netherland) detected changes in current due to the NTP treatment. In addition, the amount of eNO increased with the NTP treatment time in both media (DW and DMEM 10% FBS). eNO was formed in the range of 394, 616, and 643 nM in DW and of 127, 274, and 360 nM in DMEM 10% FBS after 10, 20, and 30 s of NTP treatment, respectively (Fig. 2c and d).

As shown in Fig. 2c and d, the quantitative results *in vitro* showed that the eNO production in DW was higher than that in DMEM 10% FBS. This result may have been due to the many molecules, such as proteins, contained in the cell culture medium, which can play the role of a NO scavenger and remove a significant amount of NO generated by the NTP treatment.

The direct quantitative results of the *in vivo* experiment using the electrochemical NO microsensor showed that the eNO produced by NTP treatment could penetrate the layers of the skin. Interestingly, even a very short period of topical NTP application (30 s) increased the amount of eNO in the dermis of the mouse skin. These *in vivo* results show, for the first time, that it is possible to perform real-time detection of eNO by NO microsensor in the skin of live animals. It was also demonstrated that NTP can be used to deliver eNO transdermally.

Based on these results, we showed that transdermal treatment using our NTP device may be a useful tool for delivering eNO to the deep dermal areas without chemical NO donors.

### *In vitro* cytotoxicity and proliferation of skin cells

**Fig. 3:**
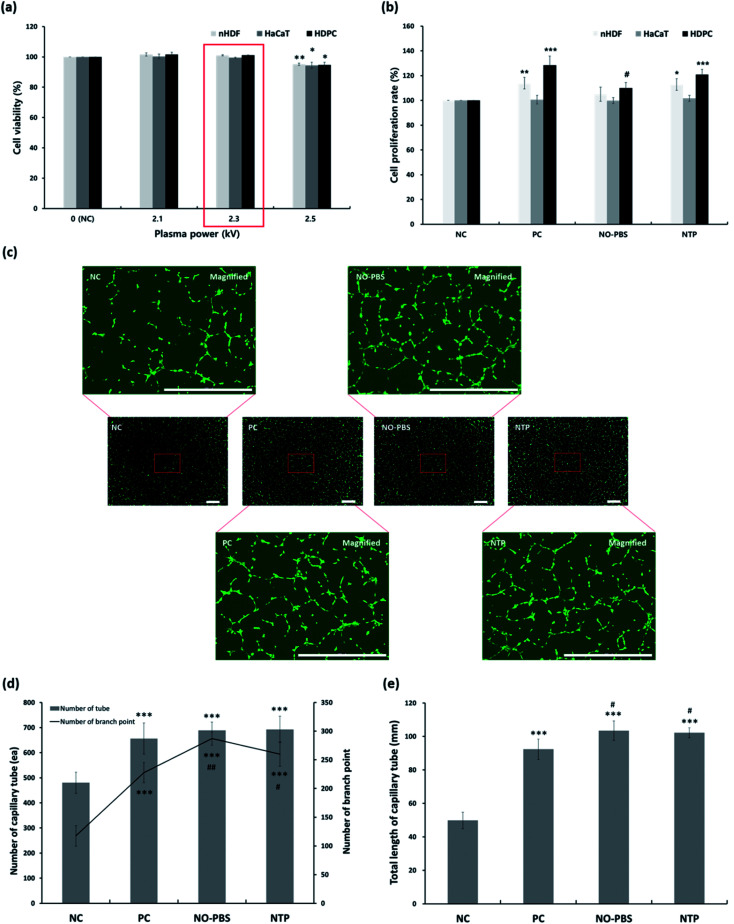
*In vitro* cytotoxicity, cell proliferation, and differentiation (capillary tube formation) assays in human skin cells. (a) Cytotoxicity depending on the NTP power. The cell viability was evaluated with a CCK-8 assay kit after incubation for 24 h, following treatment with NTP for 30 s. Data are represented as the mean ± SD (*n* = 4, *p < 0.05, **p < 0.001 *vs.* NC). (b) The proliferation of each cell after NTP treatment. Proliferation rate was detected with a CCK-8 assay kit 5 days post-treatment with NTP (2.3 kV) for 30 s. Data are expressed as the mean ± SD (*n* = 4, *p < 0.05, **p < 0.005, and ***p < 0.001 *versus* NC; ^#^*p* < 0.001 *versus* PC). (c) Capillary tube formation by minoxidil, NO-PBS, and NTP treatment in HUVECs. Each magnified image was enlarged in the middle of 5 × 5 arrays to check the conditions of capillary tubes and branch points. Scale bars are 1000 μm. (d) The number of capillary tubes and branch points and (e) the total length of capillary tubes. For the capillary tube formation assay, the HUVECs were incubated for 8 h after treatment with NTP (2.3 kV) for 30 s. The images were collected using an automated live-cell imager with a 4× objective. For the image montage, 25 images (5 × 5 arrays) were taken and selected with tile overlapping set to auto for stitching using Gen5 software. The number of capillary-like tubes, branching points, and the total length of the capillary tubes were analyzed using ImageJ software. Data are expressed as the mean ± SD (*n* = 4) and the significance was defined as ***p < 0.001 *versus* NC; ^#^*p* < 0.05 and ^##^*p* < 0.001 *versus* PC. NC: negative control, PC: positive control (minoxidil), NO–PBS: PBS solution including NO, and NTP: non-thermal plasma.

To assess changes in the cellular viability related to the percutaneous application of NTP, three types of human skin cells—keratinocytes, fibroblasts, and dermal papilla cells—were used to investigate cytotoxicity and cell proliferation (Fig. 3a and b).

In the cytotoxicity test, the NTP treatment at different output powers (2.1, 2.3, and 2.5 kV) had no cytotoxic effect on the three types of skin cells (HaCaT, nHDF, and HDPC) compared to each control cell. The average cell viabilities of HaCaTs, nHDFs, and HDPCs were greater than 94%. However, at the maximum power (2.5 kV) of the NTP application, the viabilities were slightly decreased in all of the cell types (Fig. 3a). Therefore, we chose the NTP output power to be 2.3 kV for all further experiments *in vitro* and *in vivo*.

Subsequently, we further tested the long-term cell proliferation effect of the NTP treatment using three types of skin cells (HaCaT, nHDF, and HDPC) for 5 days (Fig. 3b). The NTP treatment for cell proliferation showed similar effects of PC (minoxidil)—or rather, the positive effects of the NO solution group—although there were slight differences for each cell. In particular, the proliferation of nHDF and HDPC was significantly increased after NTP treatment. Taken together, *in vitro* cytotoxicity and cell growth analysis using human skin cells revealed NTP treatment to be a useful tool to apply to skin without causing severe cytotoxicity.

### *In vitro* capillary formation by NTP treatment

ROS and RNS can stimulate vascularization by inducing the release of pro-angiogenic growth factors, particularly VEGF and FGF-2.^37,39–42^ Therefore, we evaluated the angiogenic effect of NTP treatment by examining the capillary formation using HUVECs after staining them with calcein AM to better visualize the capillary tube formation through fluorescence imaging. In the NC group, only short or disconnected capillaries were identified during the experiment (Fig. 3c). The HUVECs in the NTP treatment, PC, and NO-PBS groups showed well-aligned capillary-like structures (Fig. 3c) and a significantly increased total branching length (Fig. 3d and e) than the NC group. NTP treatment can promote the proliferation of endothelial cells and increase angiogenesis or neovascularization.^32,38,39^ These results suggest that NTP treatment may stimulate HUVECs to increase angiogenesis *in vitro*.

### *In vitro* antibody array for hair-growth- and angiogenesis-related proteins and ELISA analysis for IGF-1 and β-catenin

**Fig. 4:**
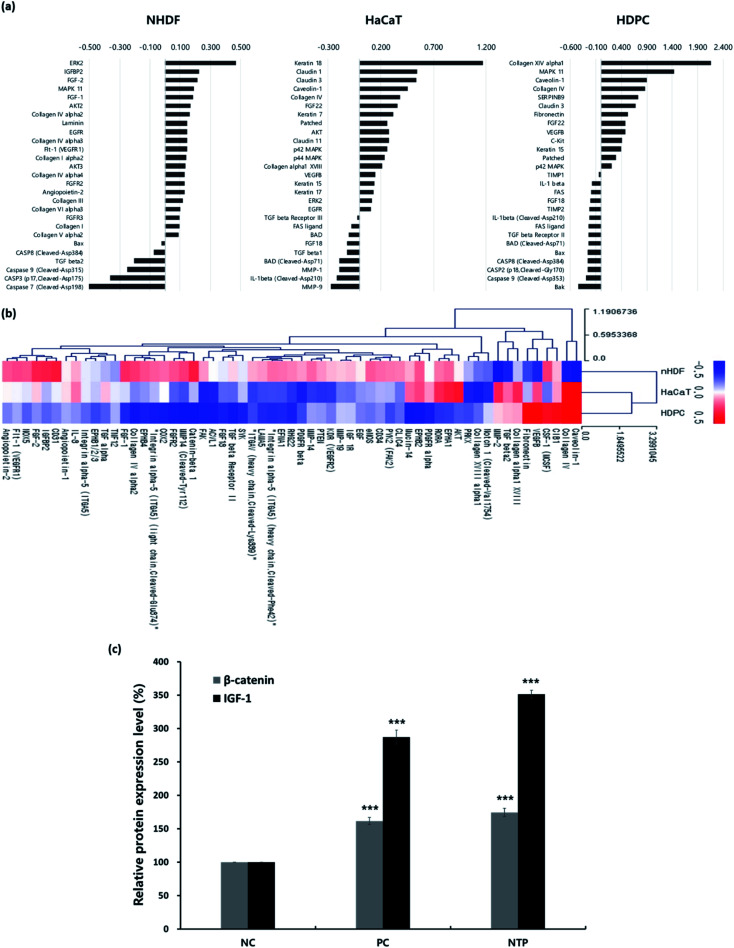
*In vitro* antibody array for differential expression of proteins related to hair-growth and angiogenesis and enzyme-linked immunosorbent assay (ELISA) analysis of β-catenin and IGF-1 expressions induced by NTP treatment. (a) Differentially expressed proteins related to hair growth, and (b) clustering heat map showing expression of proteins related to angiogenesis induced by the NTP treatment in HaCaT, nHDF, and HDPCs. The expressed hair-growth related proteins from the three cells were detected using an antibody microarray assay kit after 24 h of incubation following treatment with NTP for 30 s. Data were analyzed using Genowiz 4.0, and the data on protein information were annotated using the UniProt database. (c) ELISA analysis of β-catenin and IGF-1 in HDPCs cultured medium. Protein expression level in HDPCs cultured medium was detected after 48 h of incubation following treatment with the NTP (2.3 kV) for 30 s. The intensity of each band is represented as the mean ± SD (*n* = 4, ***p < 0.001 *versus* NC). NC: negative control, PC: positive control (minoxidil), and NTP: non-thermal plasma.

To confirm the effect of NTP treatment on hair growth, the expression levels of proteins related to hair growth and angiogenesis were investigated, as shown in Fig. 4a, b and Table 2.

**Table 2:** Summary of differential expressed proteins for angiogenesis

Cell type	Up-regulated genes	Down-regulated genes	Total
nHDF	42	17	59
HaCaT	19	40	59
HDPC	9	50	59

First, antibody microarray assays were performed using HaCaTs, nHDFs, and HDPCs to investigate the differential protein profiles after 24 h of incubation following NTP treatment. HaCaTs showed increased expression of keratin 7, 15, 17, and 18. Keratin 15, which is considered to be an HF stem cell marker, increased in both HaCaTs and HDPCs.^43^ Several types of claudin also increased in the HaCaTs, which are known to consist of tight junctions within the epidermis.^44^ As for the nHDFs, several collagens including types I, III, IV, V, and VI increased. Among these collagen types, collagen IV, often found in the basement membrane of the outer root sheath and in the extracellular matrix of the dermal papilla during the anagen and catagen phases,^45^ also increased in the HDPCs.

Moreover, proteins associated with the MAPK/ERK pathway increased in all cell types, and this could be related to the growth factors, such as fibroblast growth factors (FGFs) and VEGFs, that also increased. The expression of FGF1 and FGF2, well-known angiogenic factors, was upregulated in nHDFs along with FGF receptors, FGFR2 and FGFR3.^46^ VEGF-B was upregulated in HaCaT and HDPCs, and VEGFR1 was also upregulated in nHDFs. VEGF-B regulates cell survival, and although it may not be a direct regulator of angiogenesis, it can affect VEGF-A action; moreover, it regulates vascular survival activity, thereby indirectly promoting angiogenesis.^47,48^ Along with FGFs and VEGF-B, the expression of IGFBP2 increased in nHDFs, supporting the angiogenic effect of the NTP treatment. In addition, pro-apoptotic factors including caspases (caspase 2, 3, 7, 8, and 9), the Bcl-2 family (BAD, Bax, and Bak), and Fas and Fas ligand decreased in all cell types. NTP treatment also downregulated TGF-β1, TGF-β2, IL-1β, and FGF18, which are factors that negatively regulate hair growth.^49–51^

The HF undergoes a cyclic transformation during its maintenance, from the growth (anagen) phase to the regression (catagen) phase and the resting (telogen) phase. During the telogen-to-anagen transition, signals from the dermal papilla cells, such as synchrony Wnt/β-catenin signaling, activate stem cells and trigger coordinated epithelial–mesenchymal interactions.^52^ To analyze the mechanisms affecting hair growth by the NTP treatment, the expressions of IGF-1 and β-catenin were confirmed in HDPC using ELISA analysis after treatment with the NTP and culture for 48 h. In ELISA analysis, the expressions of both proteins, IGF-1 and β-catenin, were more significantly increased by the NTP treatment than those of the NC and PC, as shown in Fig. 4c. These results suggest that NTP treatment triggers the activation of IGF-1 and β-catenin signaling, which can stimulate hair growth.

### Effect of eNO on co-culture of HDPCs and HaCaTs

**Fig. 5:**
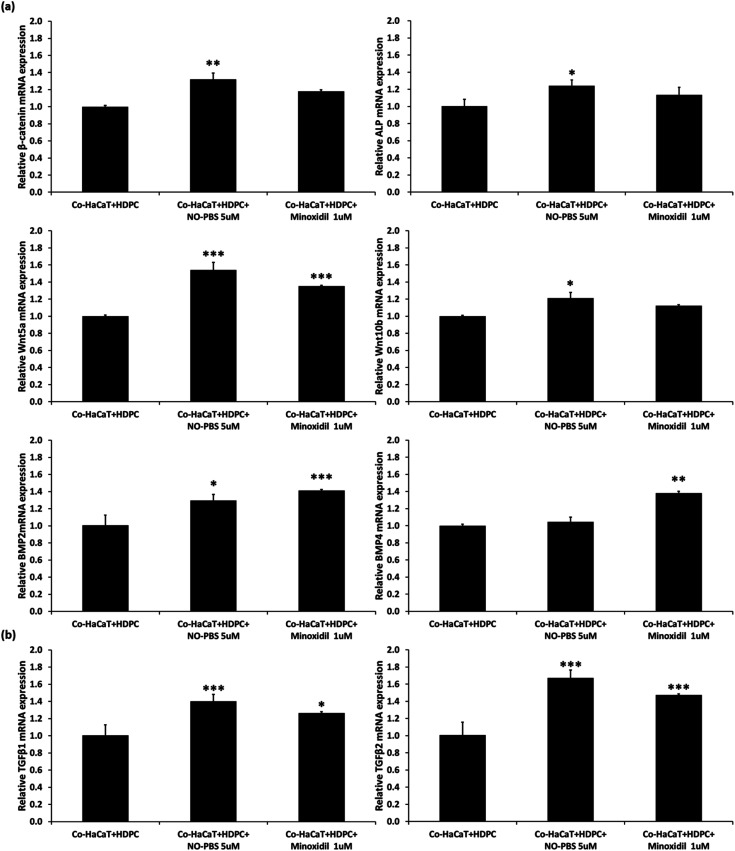
Effect of NO-treatment on hair growth in HF cells. Co-cultured HaCaT and HDPCs were treated with 5 μM NO–PBS and 1 μM minoxidil for three days. (a) Expression levels of the signature gene of dermal papilla cells were analyzed using RT-PCR. (b) mRNA expression of hair cycle-associated genes. mRNA levels were normalized with the values of reference genes, and the data were represented as fold changes of the threshold cycle (Ct) value relative to NC using the 2^−ΔΔCt^ method. Data are the mean ± SD of triplicate experiments. *p < 0.05, **p < 0.01, ***p < 0.001 *versus* NC (Co-HaCaT + HDPC); NC: negative control.

To verify the efficacy mechanism of the NTP treatment, a co-culture of HDPCs and HaCaTs was treated with 5 μM of NO-PBS. After 3 days of NO-treatment, the co-culture cells showed significantly upregulated gene expression of signature genes of the dermal papilla cell (ALP, Wnt5a, and Wnt10b). In addition, compared to the NC group, the NO-treated group showed significant upregulation of β-catenin mRNA expression, which is involved in the proliferation, differentiation, and stemness of dermal papilla cells (Fig. 5a). TGF-β1 and TGF-β2, which are known to help improve the hair cycle, were also upregulated in the NO-treated group (Fig. 5b). Interestingly, the NO-treatment group showed similar or slightly increased levels of mRNA expression of hair growth-related genes compared to 1 μM minoxidil (PC). These results suggest that the stimulating effect of NTP treatment on hair growth occurs *via* the eNO generated in the culture media.

### *In vivo* hair-growth study

**Fig. 6:**
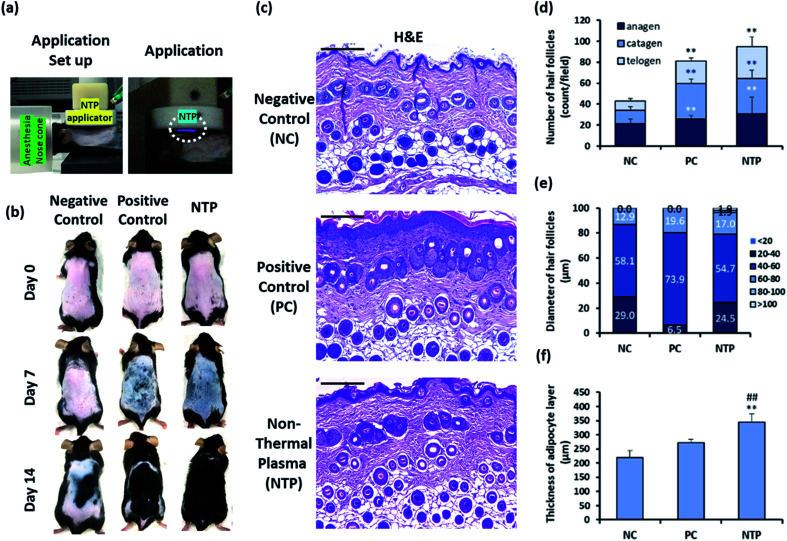
Effect of NTP treatment on clipping/hair removal cream-induced HF synchronization model. (a) NTP application set up and application moments. To clarify the visualization of the NTP (right), the pictures were taken in the dark. (b) The mice were treated with NTP (2.3 kW for 30 s). NTP treatment was performed three times per week for two weeks. For the positive control, 3 wt% minoxidil (200 μL) was applied five times per week. Representative images of the back skin were acquired on days 0, 7, and 14 after treatment (*n* = 5/group). (c) Representative images of histological sections of the skin stained with hematoxylin–eosin. (d) The number of hair follicles in each hair-growth cycle. (e) Diameter of hair follicles in the anagen phase. (f) The thickness of the adipocyte layer. Scale bars are 200 μm. *p < 0.05, **p < 0.01 *versus* NC; ^##^*p* < 0.01 *versus* PC. NC: negative control, PC: positive control (minoxidil), and NTP: non-thermal plasma.

The HF unit is a compound organ consisting of epithelial compartments (bulge stem cells, hair germ cells, and hair matrix cells) and mesenchymal compartments (dermal papilla cells and dermal sheath cells). Cyclic changes in the HF involve epithelial–mesenchymal interactions (EMIs).^52–55^ Moreover, the entire length of the telogen-to-anagen transition of the HFs resides above the dermal-adipose boundary; the adipose tissue expands as the HFs enter the anagen phase and then thins during the telogen phase through the proliferation and differentiation of the resident adipocyte precursor cells.^6,34,35^ To investigate the effect of the NTP treatment on hair growth *in vivo*, the clipping/hair removal cream-induced HF cycle-synchronized C57BL/6 mice model was used.^27,34,35^ The mice were treated with NTP or 3% minoxidil (PC) (Fig. 6a). After 7 days of treatment, the skin color in the NTP and the PC group changed from pink to black, while the NC group showed no color change (Fig. 6b). As black pigmentation on the skin represents telogen-to-anagen transition,^27,34,35^ the observation shows that NTP treatment facilitated the hair-growth cycle. On day 14, the skin was covered with fully re-grown hair in the NTP group and PC group, but the hair grew only in limited areas in the NC group (Fig. 6b).

Histological analysis showed that the NTP group had a significantly increased total number of HFs compared to the NC and PC groups (Fig. 6c and d). The increase in the NTP-treated group was 220.4% ± 38.3% greater than the NC group and 116.8% ± 20.3% greater than the PC group (Fig. 6d). Furthermore, the number of anagen follicles in the NTP-treated group increased by 179.3% ± 36.2% compared to the NC group (Fig. 6d).

Notably, the thickness of HFs in the anagen phase differed between groups (Fig. 6e). The percentage of HFs over 60 μm in diameter increased due to NTP treatment and in the PC group, as compared to the NC group. Moreover, HFs with a diameter of over 80 μm were observed only in the NTP-treated group. The effects of NTP treatment on hair growth were further examined by measuring the thickness of the dermal adipocyte layer. At the initiation of the anagen phase, the HFs grow deeply into the dermal adipocyte layer and the thickness of the dermal adipocyte layer dramatically increases.^56,57^ Recent advanced studies have revealed the importance of adipocytes in hair regeneration and actual changes in the number of adipocyte precursor cells.^53^ Moreover, adipogenesis and the changes in the adipocyte layer show synchrony with the activation of follicular stem cells and HF cycling.^58,59^ The changes in the thickness of the dermal adipocyte layer exhibit a synchronized cycle with HF regeneration.^60^ The results showed that the thickness of the dermal adipocyte layer increased due to NTP treatment and that it was significantly thicker in the NTP treatment group than in the PC and NC groups (Fig. 6f). Taken together, these results suggest that the application of NTP not only induces an increase in the anagen phase/total number of HFs but also provides an HF regenerating macroenvironment by enriching the adipocyte layer.

### *In vivo* evaluation of perifollicular vessels

**Fig. 7:**
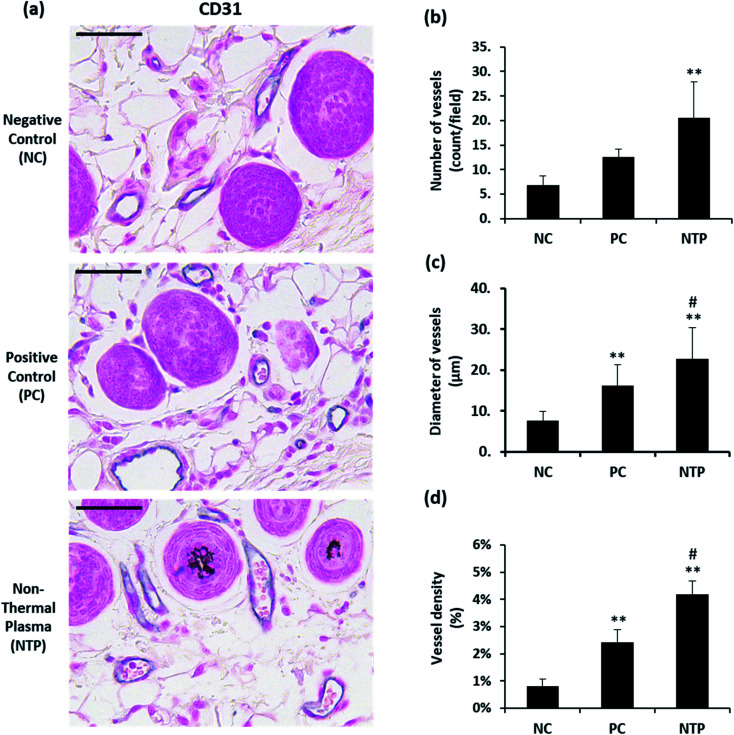
Effect of NTP treatment on perifollicular vessels. (a) Representative images of immunohistochemical staining with anti-CD31 antibody. (b) Number of vessels and (c) diameter of vessels in multiple fields at 100× magnification. (d) Vessel density was determined by the area (%) covered with vessels at 100× magnification. Scale bars are 100 μm. **p < 0.01 *versus* NC; ^#^*p* <0.05 *versus* PC. NC: negative control, PC: positive control (minoxidil), and NTP: non-thermal plasma.

One of the important macroenvironment elements related to HFs is the perifollicular vessel. HFs are avascular structures, but they are surrounded by perifollicular vessels, which are connected to the vascular plexus. The perifollicular vessels are essential for growing HFs and for their maintenance by providing oxygen and nutrients.^7^ Perifollicular vessels become more prominent when HFs are in the anagen phase, providing nutrition as they undergo rapid proliferation and morphogenesis.^61^ To examine whether NTP treatment enhances angiogenesis, the number, diameter, and density of perifollicular vessels were assessed. The skin sections were stained with CD31, an endothelial junction molecule, to observe the blood vessels (Fig. 7a). The results revealed a more than two-fold increase in the number and diameter of perifollicular vessels in the NTP-treated group (Fig. 7b). The increase in diameter in the NTP-treated group was more prominent than that in the PC group (Fig. 7c, *p* < 0.05). Vascular density was determined as the area covered with vessels in multiple fields at 100× magnification (Fig. 7d). The NTP-treated group and the PC group showed an increase in vessel density. Notably, the increase in the NTP-treated group was more than five times that in the NC group, showing the highest density. Our results show that NTP treatment can improve the perifollicular blood supply and increase the HF diameter. NTP treatment had profound effects on the blood supply, as demonstrated by the number, diameter, and density of the vessels, which was greater than that following minoxidil treatment. Given that minoxidil, which was used as a PC, is well-known to stimulate peripheral vasodilation and circulation, these results indicate that NTP treatment can improve blood supply to the HFs as much as that afforded by minoxidil.

### *In vivo* examination of IGF-1 and β-catenin expression

**Fig. 8:**
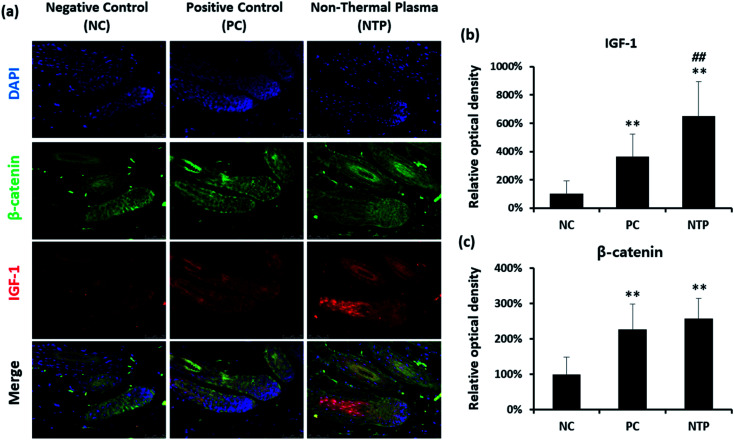
Effect of NTP treatment on IGF-1 and β-catenin level. (a) Double immunofluorescence with antibodies against β-catenin (green) and IGF-1 (red). (b) Relative optical density of IGF-1 and (c) β-catenin in hair follicles. **p < 0.01 *versus* NC; ^##^*p* < 0.05 *versus* PC. NC: negative control, PC: positive control (minoxidil), and NTP: non-thermal plasma.

To further identify the hair-growth-promoting effect of NTP treatment, IGF-1 and β-catenin expressions were examined (Fig. 8). The expression of IGF-1 on HFs was upregulated in both the NTP and PC groups. Interestingly, the results from the NTP-treated group were significantly higher than those of the other groups (Fig. 8b). The β-catenin level was also increased in both treatment groups (Fig. 8c). These results correlate with the *in vitro* results (Fig. 4c) and suggest that the increased hair growth induced by NTP treatment upregulated IGF-1 signaling and promoted the Wnt/β-catenin signaling pathway.

### *In vivo* evaluation of stem cell activity by RNA *in situ* hybridization

**Fig. 9:**
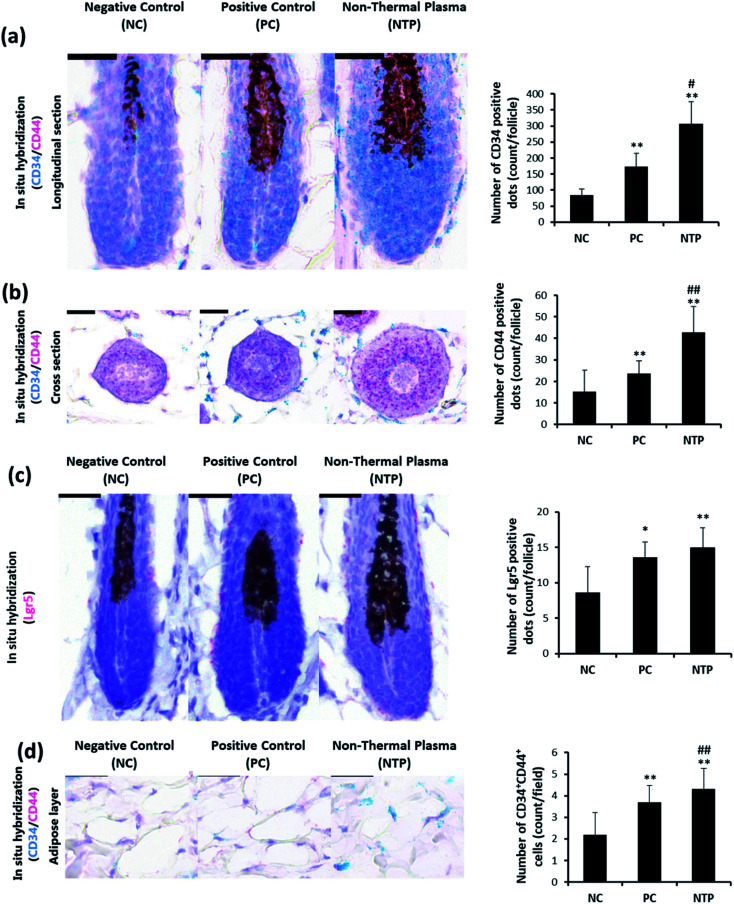
Effect of NTP treatment on CD34, CD44, and LGR5 RNA expressions. (a–c) Representative images of RNA *in situ* hybridization results in hair follicles in a longitudinal section and cross-section with probes against CD34 (green), CD44 (red), and LGR5 (red). (d) Representative image of CD34^+^CD44^+^ cells in the adipose layer. Scale bars are 30 μm. **p < 0.05 *versus* NC; ^##^*p* < 0.01 *versus* PC. NC: negative control, PC: positive control (minoxidil), and NTP: non-thermal plasma.

To determine whether the increased IGF-1 level and β-catenin signaling are mediated by increased stem cell activity in adipose tissue, CD34, CD44, and LGR5 expressions were examined by RNA *in situ* hybridization. As shown in Fig. 9a, the anagen follicles in adipose tissue at the mid-bulb level showed a marked CD34 expression on the outermost region of the HF where the bulge stem cells are located.^62,63^ The CD34 and LGR5 expressions were significantly increased with NTP treatment, demonstrating increased bulge stem cell activity (Fig. 9a and c). The CD44 expression was also increased at this level and was more evident in follicles at the lower bulb level (Fig. 9a and b). The markedly increased expression of CD44 at the lower bulb, where active progenitor cells are located, also suggests increased stem cell activity (Fig. 8b).^64–68^ CD34 and CD44 are expressed in mesenchymal stem cells;^69,70^ thus, we further examined their expressions in the adipose tissue. The results showed that the number of CD34^+^ CD44^+^ cells increased with NTP treatment (Fig. 9d). This result, together with the increased thickness of the adipocyte layer, confirmed that NTP treatment stimulated inter-follicular adipogenesis.

## Conclusions

Currently, various types of laser-based devices are being utilized in clinical practice as an alternative treatment for hair growth, and laser-based devices have been shown to increase blood supply, signaling molecules, and growth factors for hair follicles.^12–15^ In particular, low-level lasers have been shown to be useful for the treatment of hair loss, and some of them are commercially available at present. However, certain types of lasers such as fractional lasers are relatively expensive, and some treatments have been shown to induce side effects along with burns or microscopic injuries on the patient's skin owing to their strong energy.^16^

Meanwhile, advances in the NTP device and its application can greatly benefit the control of various skin conditions, including hair loss. Although it is not a life-threatening disease, hair loss affects one's self-esteem and may thereby cause social problems. NTP treatment is an easy-to-use, non-invasive method that can also be developed as a wearable device, for example, as an attachable pad.^32^ Moreover, NTP devices are easier to manufacture than lasers, are simple to use, and have the advantage of being portable due to their small size as shown in Fig. 1b. In addition, NTP treatment not only activates skin cells without causing any damage, unlike that in the case of laser, but also allows transdermal NO generation to promote hair growth through vascularization, adipogenesis, and stem cell activation. It is also known to be effective against bacterial and fungal skin diseases owing to its antibacterial effect against various skin bacteria.

For these reasons, we examined the effect of NTP treatment on hair growth by demonstrating its effect on the microenvironment of HFs and the inter-follicular dermal macroenvironment.

Our study suggests, for the first time, that NTP treatment efficiently increases the amount of transdermal eNO in the mouse skin without causing any damage, resulting in significant promotion of hair growth by improving IGF-1 and β-catenin levels in the HFs and stem cell activity, as well as in the improvement of the inter-follicular macroenvironment by enhancing perifollicular vascularity and adipogenesis. Therefore, we propose that NTP may be a promising alternative therapy in regenerative medicine to safely deliver eNO for the treatment of hair loss disorders without requiring any additional drug or material.

Nevertheless, clinical trials are warranted to confirm this hypothesis. The biological effects of eNO produced from NTP toward hair growth must be studied deeply to obtain a better understanding of its role in the skin and its microenvironments.

## Author contributions

The manuscript was written with contributions from all authors. All authors approved the final version of the manuscript. In detail, Guangsup Cho, Sun Hee Do, and Bong Joo Park conceived and designed all the experiments. Junggil Kim, Guangsup Cho, Eun-Wook Choi, and Bong Joo Park designed, fabricated, and characterized the non-thermal plasma device. They wrote the original draft for the plasma device. Han-Jun Kim, Eun-Wook Choi, Eun-Ji Choi, Hyo-Sung Kim, Sun Hee Do, and Bong Joo Park conducted *in vitro* cell works and *in vivo* animal experiments and wrote the methodology and original draft for the manuscript. Heesu Kim, Seulgi Na, and Jae Ho Shin performed *in vitro* and *in vivo* experiments for nitric oxide detection using an electrochemical sensor and analyzed the data. They also wrote the original draft. Sun Hee Do and Bong Joo Park reviewed and edited the original draft.

## Conflicts of interest

There are no conflicts to declare.

## Supplementary Material
